# Can EGCG Alleviate Symptoms of Down Syndrome by Altering Proteolytic Activity?

**DOI:** 10.3390/ijms19010248

**Published:** 2018-01-15

**Authors:** Marzena Wyganowska-Świątkowska, Maja Matthews-Kozanecka, Teresa Matthews-Brzozowska, Ewa Skrzypczak-Jankun, Jerzy Jankun

**Affiliations:** 1 Department of Dental Surgery and Periodontology, Poznań University of Medical Sciences, Bukowska 70, 60-812 Poznań, Poland; 2 Department of Social Sciences, Poznań University of Medical Sciences, Fredry 10, 61-710 Poznań, Poland; pszczolka-maja@o2.pl; 3 The Chair and Clinic of Maxillofacial Orthopaedics and Orthodontics, Poznań University of Medical Sciences, Bukowska 70, 60-812 Poznań, Poland; teresamatbrzo@gmail.com; 4 Urology Research Center, Department of Urology, The University of Toledo-Health Science Campus, 3000 Arlington Ave, Toledo, OH 43614, USA; Ewa.Skrzypczak-Jankun@utoledo.edu (E.S.-J.); Jerzy.Jankun@utoledo.edu (J.J.)

**Keywords:** Down syndrome, epigallocatechin gallate, metalloproteinase 9, plasminogen activator system

## Abstract

Down syndrome (DS), also known as “trisomy 21”, is a genetic disorder caused by the presence of all or part of a third copy of chromosome 21. Silencing these extra genes is beyond existing technology and seems to be impractical. A number of pharmacologic options have been proposed to change the quality of life and lifespan of individuals with DS. It was reported that treatment with epigallocatechin gallate (EGCG) improves cognitive performance in animal models and in humans, suggesting that EGCG may alleviate symptoms of DS. Traditionally, EGCG has been associated with the ability to reduce dual specificity tyrosine phosphorylation regulated kinase 1A activity, which is overexpressed in trisomy 21. Based on the data available in the literature, we propose an additional way in which EGCG might affect trisomy 21—namely by modifying the proteolytic activity of the enzymes involved. It is known that, in Down syndrome, the nerve growth factor (NGF) metabolic pathway is altered: first by downregulating tissue plasminogen activator (tPA) that activates plasminogen to plasmin, an enzyme converting proNGF to mature NGF; secondly, overexpression of metalloproteinase 9 (MMP-9) further degrades NGF, lowering the amount of mature NGF. EGCG inhibits MMP-9, thus protecting NGF. Urokinase (uPA) and tPA are activators of plasminogen, and uPA is inhibited by EGCG, but regardless of their structural similarity tPA is not inhibited. In this review, we describe mechanisms of proteolytic enzymes (MMP-9 and plasminogen activation system), their role in Down syndrome, their inhibition by EGCG, possible degradation of this polyphenol and the ability of EGCG and its degradation products to cross the blood–brain barrier. We conclude that known data accumulated so far provide promising evidence of MMP-9 inhibition by EGCG in the brain, which could slow down the abnormal degradation of NGF.

## 1. Introduction

Down syndrome (DS), also known as “trisomy 21”, is a genetic disorder caused by the presence of all or part of a third copy of chromosome 21 [1,2]. Chromosome 21 is the smallest human chromosome, containing approximately 48 million nucleotides (~1.5% of the cellular DNA). It is estimated that this part of the genome encodes for 127 known genes, 98 predicted genes and 59 pseudogenes [3]. Silencing these extra genes is beyond existing technology and seems to be impractical. However, alternative medical techniques have been proposed such as dietary changes, massage, animal therapy, chiropractic treatments and naturopathy, among others. Unfortunately, they are poorly supported by evidence [4,5,6]. One other alternative treatment that is gaining popularity and is supported by some evidence of efficacy is green tea extract containing EGCG (epigallocatechin-3-gallate) [7]. De la Torre et al. enrolled adults with Down syndrome in a double-blind, placebo-controlled trial to receive EGCG (9 mg/kg of body weight, per day, in greet tea extract standardized for EGCG). The authors stated that this trial shows improvement in the adaptive behavior and brain-related changes in young adults with Down syndrome [7]. They attribute the effectiveness of EGCG to its ability to cross the blood–brain barrier and noncompetitive inhibition of tyrosine-(Y)-phosphorylation-regulated kinase 1A encoded by the *DYRK1A* gene, located on the long arm of chromosome 21 and postulated to be a key contributor to cognitive phenotypes of Down syndrome [7].

It was reported that the nerve growth factor (NGF) metabolic pathway in Down syndrome is altered. While proNGF is secreted in the extracellular space, the zymogens, enzymes and regulators necessary for its maturation and degradation are released as well. This includes tissue plasminogen activator (tPA), plasminogen and matrix metalloproteinase 9 (MMP-9). Tissue plasminogen activator activates plasminogen to strong proteolytic enzyme–plasmin, and plasmin converts proNGF to mature NGF. The inhibitor of tPA, neuroserpin, regulates the proNGF maturation in the central nervous system by suppressing tPA activity. Mature NGF can bind receptors—tropomyosin receptor kinase A (TrkA)/p75 neurotrophin receptor (p75NTR)—or else it is degraded by the matrix metalloproteinase 9. In Down syndrome, the availability of mature NGF is compromised by diminished tPA/plasminogen → plasmin activity, which limits the production of NGF, further lowered by the increased activity of MMP-9 that degrades NGF [8,9].

EGCG can inhibit/activate numerous pathways and proteins, for example, urokinase and metaloproteinases such as MMP-9 [10,11]. It was reported that in brains from individuals with DS, tPA-driven proteolytic activity is altered and zymogenic MMP-9 activity is elevated [9]. Thus, in this paper, we review the role of proteolysis in Down syndrome and a possible role of EGCG in proteolysis alternation.

## 2. The Plasminogen Activator System (PAS)

**Figure 1:**
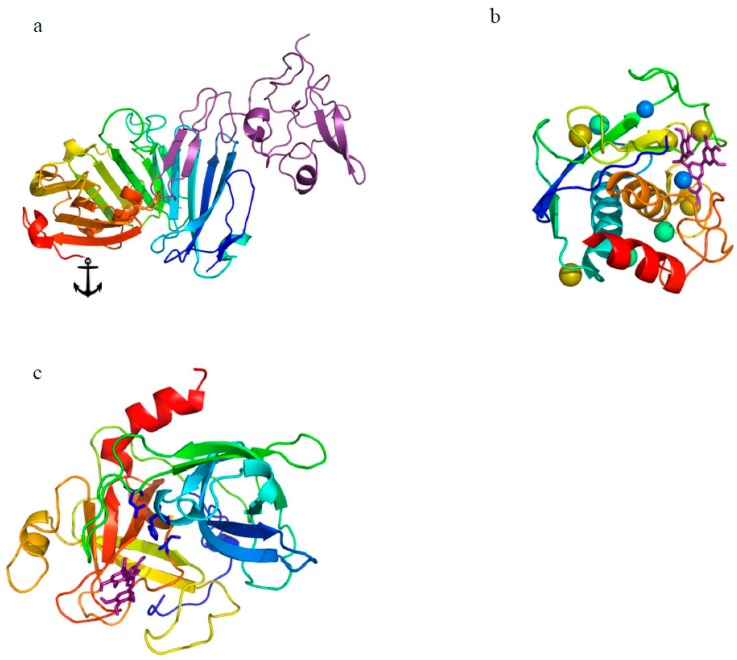
Illustration of proteins. (**a**) amino-terminal fragment of urokinase plasminogen activator (uPA) binds to the central cavity of soluble urokinase-type plasminogen activator receptor (suPAR) shown in purple. uPAR domains (Protein Data Bank entry: 3U73): residues 1–93 domain DI shown in blue–light blue, residues 94–191 domain DII shown in green, yellow–green, residues 192–277 domain DIII shown in light-brown, red; uPAR is attached to the cell surface by a GPI-anchor (glycosylphosphatidylinositolat) of the D-III domain, protein structure (Protein Data Bank entry: 2I9B) [23], (**b**) MMP-9 in complex with epigallocatechin gallate where EGCG is shown in purple as a stick model; ions in MMP-9 are shown as spheres: Zn^2+^ in blue, Ca^2+^ in yellow, Cl^−^ in green [10], MMP-9 protein structure (Protein Data Bank entry: 2OVX) [24], (**c**) EGCG is shown as a stick model in purple bound to the specificity pocket of urokinase close to the catalytic triad shown as sticks in blue [10]; protein structure (Protein Data Bank entry: 2VNT) [25]. All drawings made using program PyMOL

PAS consists of several proteins: (i) two serine proteases, the urokinase plasminogen activator (uPA) and the tissue-type plasminogen activator (tPA), that activate plasminogen to serine protease called plasmin, which is able to lyse a wide range of proteins including laminin, vitronectin, type IV collagen, and proteoglycans; (ii) activator inhibitors such as the plasminogen activator inhibitor 1 (PAI-1) and the less common PAI-2 and protease nexin-1 (PN-1); (iii) cell membrane anchored receptor for the uPA that localizes proteolytic activity in the proximity of the cell membrane (Figure 1). Historically, plasmin was recognized as instrumental in fibrin degradation during clot lysis. However, later, it was found to be involved in a number of physiological and pathological processes such as extracellular matrix (ECM) and basement membrane (BM) remodeling, mammary gland development, lactation, wound healing, angiogenesis, tumor progression, invasion, and metastasis [9,12,13,14,15,16]. Moreover, plasmin may also activate other latent proteases such as matrix metalloproteinases (MMPs) and collagenases [17]. Hepatocyte growth factor/scattering factor (HGF/SF) belongs to the plasminogen activation system but does not have any proteolytic activity. It is secreted by mesenchymal cells as a single inactive protein, and is cleaved by serine proteases into 69-kDa α-chain and 34-kDa β-chain connected by a disulfide bridge, producing fully active molecules [18,19,20,21]. Maturation of this inactive protein into the active form was reported in vitro in the presence of nanomolar concentrations of uPA. This cleavage was prevented by urokinase inhibitors, such as PAI-1, and by antibodies specific for the uPA catalytic domain [22].

## 3. Matrix Metalloproteinases

Matrix metalloproteinases are a large group of proteins (from MMP-1 to MMP-28) that are metal-dependent (calcium or zinc) endopeptidases [26]. These enzymes are degrading extracellular matrix proteins, and can process a number of proteins, cleaving cell surface receptors, and releasing apoptotic ligands. In that way, MMPs play a major role in cell differentiation, migration, proliferation, angiogenesis, and apoptosis, in normal physiological and pathological processes [27]. MMP-9 (known as 92 kDa type IV collagenase, or gelatinase), belongs to a class of enzymes that is the zinc-activated family of the extracellular matrix lysing proteins. MMP-9 is involved in embryonic development, reproduction, angiogenesis, bone development, wound healing, cell migration, learning and memory, and some pathological processes (arthritis, intracerebral hemorrhage, and metastasis) [28,29]. MMP-9 is recognized as an important enzyme because of its role in various types of diseases, such as cancer and progressive proliferative vascular disorder. Progressive proliferative vascular disorder results from persistent vasoconstriction associated with the activation of MMP [30]. The activation of MMPs starts at the gene transcription level, followed by posttranslational activation of zymogens by the loss of a 10-kDa peptide [31,32]. The activity of MMPs is counterbalanced by α2-macroglobulin or by the tissue inhibitors of metalloproteinases (TIMPs) providing balance between production, activity, and inhibition [31,33,34].

## 4. Tissue Inhibitors of Metalloproteinases

TIMPs are specific inhibitors that bind MMPs in a 1:1 stoichiometry and contain a family of four protease inhibitors: TIMP-1, TIMP-2, TIMP-3 and TIMP-4 [35]. TIMPs bind to most MMPs but some differences in the inhibition of different TIMPs have been reported. For example, TIMP-2 and TIMP-3 are effective inhibitors of the membrane-type MMPs (MT-MMPs), while TIMP-3 is a good inhibitor of tumor necrosis factor-α converting enzyme. Unfortunately, there are little data from comprehensive evaluations of the complete interactions of different TIMPs with MMPs [36]. Nevertheless, TIMP-2 binds strongly to the proMMP-2, while TIMP-1 forms a complex with proMMP-9, and TIMP-4 binds to the C-terminal domain of proMMP-2 [37,38,39]. TIMP-3 appears to bind strongly to extracellular matrix components but is a less investigated member of the family [38,40].

## 5. Plasminogen Activator System in Down Syndrome

One of characteristics of Down syndrome is accumulation of amyloid-β peptides in brain tissue. It progressively deposits in the brains of individuals with DS from early life [41] and by middle-age almost all individuals with DS have the neuropathological hallmarks characteristic of Alzheimer’s disease [9]. Deficit of nerve growth factor (NGF) could be the possible mechanism by which PAS can impact the degeneration of neurons.

## 6. Tissue Plasminogen Activator in Down Syndrome

The proNGF is released to the extracellular space, along with the enzymes necessary for its conversion to mature NGF and for its subsequent degradation [42]. ProNGF is cleaved and matured by plasmin, which derives from plasminogen by the action of tPA. Plasmin cleaves β-amyloid at certain sites and it is known that exogenously added plasmin blocks β-amyloid neurotoxicity, supporting a physiological role for plasmin in Alzheimer’s disease [43,44,45,46]. In this context, defective amyloid peptide degradation results from a decrease in tPA expression and from an increase in the production of PAI-1, respectively, which further diminishes tPA-induced plasmin activity. Iulita et al. [9] revealed a significant increase in proNGF levels in brains from mice and individuals with DS, with a concomitant reduction in the levels of plasminogen and tissue plasminogen activator messenger RNA, as well as a boost in neuroserpin expression; enzymes that partake in proNGF maturation. Brains from individuals with DS also exhibited elevated zymogenic activity of MMP9, the major NGF-degrading protease [47,48]. At the cingulate and prelimbic cortices, the number of *cFos-*positive cells was significantly increased in tPA knockout mice compared with that in tPA Het mice after social stimulation [49]. tPA KO mice spent significantly more time undertaking active behavior, walking a greater distance in the chamber containing an empty cage, and approaching familiar and unfamiliar mice more often than tPA did [49].

## 7. Plasminogen Activator Inhibitor Type 1 in Down Syndrome

The role of plasminogen activator inhibitor 1 (PAI-1) in the brain, and the regulation of its expression in neurons are poorly understood. PAI-1 protects neuronal cell tissue injury promoted by tPA, as well as neurites of neurons, and prevents the disintegration of the formed neuronal networks by maintaining or promoting neuroprotective signaling through the mitogen-activated protein kinase/extracellular signal-regulated kinase (MAPK/ERK) pathway or phosphatidylinositol 3-kinase (PI-3 kinase) pathway, suggesting that the neuroprotective effect is independent of PAI-1 action as a protease inhibitor [50]. The other suggested pathway is via low-density lipoprotein receptor-related protein (LRP), the gene of which is located on chromosome 12, the site of potential Alzheimer’s disease locus [50]. Individuals with DS are at increased risk of developing Alzheimer’s disease as a result of triplication of the amyloid precursor protein (*APP*) gene. The increased dosage of the gene for APP (amyloid precursor protein) is linked to failed NGF signaling and cholinergic neurodegeneration in a mouse model of DS [51]. The progressive degeneration of the basal forebrain cholinergic system in both conditions may be associated with deficits of nerve growth factor (NGF), which stimulate PAI-1 mRNA expression [15,42,52]. Hypothetically, NGF induces the upregulation of PAI-1 via the calcineurin/nuclear factor of activated T cells (NFAT) pathway. Stefos et al. revealed that overexpression of the Down syndrome-related proteins encoded by gene either *DYRK1A* or *RCAN1*, negatively regulated NFAT-dependent transcriptional activity and reduced the upregulation of PAI-1 levels by NGF [42]. The authors concluded that the negative effect of *DYRK1A* and *RCAN1* overexpression on NGF signal transduction in neural cells may contribute to the altered neurodevelopment and brain function in Down syndrome [42]. Interestingly, Takahashi et al. found that genistein, an inhibitor of tyrosine protein kinase, completely inhibited NGF-induced PAI-1 mRNA in the presence of 100 µM, and wortmannin, a potent and specific inhibitor of PI-3 kinase, decreased the induction of PAI-1 mRNA level at doses of equal or greater than 10^−7^ M [15]. This suggests that both phosphorylation of the NGF receptor, Trk, and activation of the PI-3 kinase-dependent signal transduction pathway are necessary for the expression of PAI-1 mRNA in PC-12 cells [15,42,52].

## 8. Urokinase in Down Syndrome

The role of urokinase in Down syndrome is unknown. There are some possible mechanisms such as the modulation by *LRP1* (encoding low density lipoprotein receptor-related protein 1) of the axonal regeneration in ischemic stroke through the binding of uPA to uPAR in the periphery of growth cones of injured axons [53]. For Alzheimer’s disease, a number of susceptibility loci were identified, including a region on chromosome 10q21–q22. Within this region, the plasminogen activator urokinase gene (*PLAU*) was considered as a reasonable candidate from its functional implication in plasmin generation [54]. Farias-Eisner et al. [55] have demonstrated that, in NGF-induced differentiation of PC-12 cells, the expression and function of urokinase-type PA’s receptor is required transiently only during the early stages of their differentiation.

## 9. Metrics Proteins

Matricellular proteins (MCPs), non-structural proteins present in the extracellular matrix, play a major role in cell–cell interactions and tissue repair, because of binding sites for other extracellular proteins, cell surface receptors, growth factors, cytokines and proteases. The MCPs found in the brain include thrombospondin-1/2 and the secreted protein acidic and rich in cysteine (SPARC) family, both secreted from astrocytes [56]. The potential role of MCP and therapeutic opportunities for Down syndrome are probably connected with PAI-1 which, interestingly, mediates the neuroprotective activity of TGF-β1 against *N*-methyl-d-aspartate (NMDA) receptor-mediated excitotoxicity. PAI-1 protected neurons against NMDA-induced neuronal death by modulating the NMDA-evoked calcium influx [56,57]. Interestingly, the neuroprotective effect of EGCG is mediated through the reestablishment of the NMDA receptor-reactive oxygen species (ROS) system in an experimental model of Alzheimer’s disease [58].

## 10. Matrix Metalloproteinases and Down Syndrome

It has been reported that MMP-9 shows higher activity or amount in comparison with controls in many different brain conditions, including autism and Down syndrome [31,59,60,61,62,63,64]. The examination of brains from adult individuals with DS taken post mortem from the temporal, frontal and parietal cortex and brains from Ts65Dn mice (12–22 months), as well as primary cultures of cortex foetal brains from individuals with DS (17–21 gestational age weeks) revealed, similar to Alzheimer’s disease, that the synthesis of NGF is not affected and there is an abundance of the NGF precursor, proNGF. Brains from individuals with DS also exhibited elevated zymogenic activity of MMP9, the major NGF-degrading protease. Zinc-mediated extracellular activation of MMP underlies the up-regulation of brain-derived neurotrophic factor (BDNF) and the Trk signaling pathway necessary for the expression of PAI-1 mRNA [42]. Green tea polyphenols potentiated BDNF. This process requires the cell-surface-associated 67 kDa laminin receptor (67LR) to which EGCG binds with high affinity. A synergistic interaction was observed between green tea polyphenol constituents, where epigallocatechin and epicatechin, both individually lacking this activity, promoted the action of EGCG [65].

## 11. Epigallocatechin Gallate

EGCG, also known as epigallocatechin-3-gallate or (IUPAC ID: [(2*R*,3*R*)-5,7-dihydroxy-2-(3,4,5-trihydroxyphenyl)chroman-3-yl] 3,4,5-trihydroxybenzoate), is a member of the catechin family. EGCG, the ester of epigallocatechin and gallic acid, is the most abundant catechin of green tea. Because of its solubility in water and many beneficial health properties, it is under research for its potential applications for human health and against diseases, and is a common component of many dietary supplements [66,67]. What is especially appealing about this molecule is that it is safe at very high doses (up to 800 mg/day), enabling its use in many pharmaceutical applications [66]. Abundant targets for EGCG have been suggested, such as (i) PI3K/AKT (signaling pathway including phosphatidylinositol 3-kinase (PI3K), and serine/threonine kinase (AKT), also known as PKB); (ii) JAK/STAT (signaling pathway consisting of three components: a cell surface receptor, a Janus kinase (JAK) and two Signal Transducer and Activator of Transcription (STAT) proteins); (iii) MAPK (mitogen-activated protein kinase), [68], as well as (iv) proteases such as metalloproteinases and (v) urokinase [11,69,70,71]. The mechanism of action of EGCG is complicated given the fact that it is labile in aqueous solutions and modifies itself by auto-oxidization and epimerization [72].

**Figure 2:**
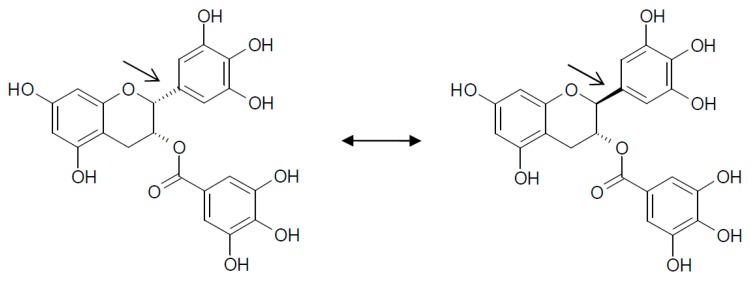
(−)EGCG can convert into (−)GCG [73,74]. Epimerization of EGCG significantly changes the three-dimensional structure of this compound that can interact differently with proteins that are relevant to Down syndrome.

EGCG is not a stable molecule in thermally processed products. For example, during sterilization of canned and bottled green tea beverages, by pasteurization at 120 °C for several minutes, considerable amounts (approximately 50%) of (−)EGCG go through epimerization (Figure 2) converting (−)EGCG to (−)GCG [73,74]. It was reported that the degradation and epimerization of EGCG happened at the same time [73]. The degradation occurred by oxidation, dimerization, and polymerization. Stability is pH and temperature dependent, EGCG in aqueous solution is stable at pH <4, and at pH >6. Moreover, at temperatures below 44 °C, the degradation of EGCG is dominant, but at temperatures higher than 98 °C, the epimerization became prominent [75].

**Figure 3:**
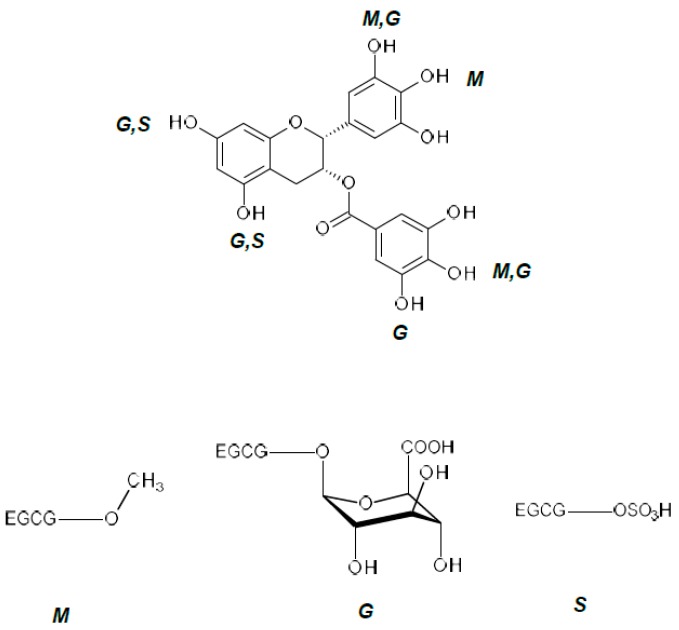
The possible sites of methylation (M), glucuronidation (G) and sulfation (S) of EGCG [89,90]. Chemical modification significantly modifies EGCG, raising questions regarding how or if these derivatives interact with proteins relevant to Down Syndrome.

Also, EGCG binds to plasma proteins, changing its plasma concentration and biological activity. Furthermore, the low systemic bioavailability of EGCG when taken orally, and its conversion to the glucuronide casts doubt on whether EGCG acts in vivo [76,77]. After oral absorption, EGCG is subject to extensive methylation, glucuronidation, and sulfation (Figure 3) [75,77,78]. Methylation decreases its hydrophilicity and further sulfation/glucuronidation leads to the elimination of methylated product from the body [78]. One of the possible solutions is to encapsulate EGCG in nanoparticles that are suitable for oral delivery, as suggested by Siddiqui et al. and Wang et al. [79,80], to increase bioavailability.

Also, metal ions affect the activity and stability of EGCG, for example, hard water containing high amounts of Ca^2+^ and Mg^2+^, or drinking milk together with EGCG inactivate it [10,11,78]. However, other studies have reported contrary opinion, stating that EGCG can be protected or activated by milk proteins [81,82,83].

One of the questions that one should ask is whether EGCG can react with proteases in vivo. In fact, it was reported that the EGCG inhibits the plasminogen activator and MMP-9 in vivo [69,84,85,86]. The inhibitory activity of EGCG was associated with the inhibition of cancer invasion by suppressing specifically the activity of urokinase or the matrix metalloproteinases (MMPs) [69,84,85,86]. Moreover, the blood–brain barrier (BBB) permeability of EGCG and its metabolites were tested by the BBB model kit. Two different reports provide evidence that EGCG, as well as its metabolites or degradation products, can reach the brain parenchyma [87,88].

**Figure 4:**
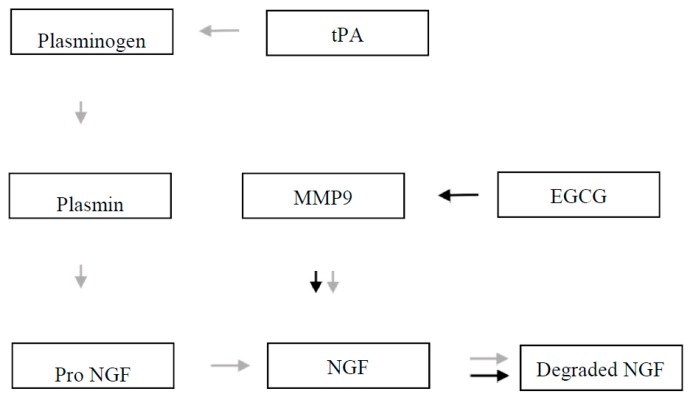
Diminished activity of tissue plasminogen activator (tPA) in Down syndrome brain converts less plasminogen to plasmin; this produces less proNGF to activate nerve growth factor (NGF) (gray arrows). Overexpressed MMP-9 further reduce NGF (black arrows). Degradation of NGF can be reduced by EGCG that inhibits MMP-9 [9,17,47].

In light of these reports, it is plausible that EGCG can be physiologically active in the brain and can modify the proteolytic activity. In Down syndrome, the nerve growth factor (NGF) metabolic pathway is altered: first by downregulating tissue plasminogen activator (tPA) that activates plasminogen to plasmin, an enzyme converting proNGF to mature NGF; secondly, overexpression of metalloproteinase 9 (MMP-9) further degrades NGF, lowering the amount of mature NGF. Thus, EGCG that inhibits MMP-9 could slow down the abnormal degradation of NGF (Figure 4). Plasminogen has two activators: urokinase (uPA) and tPA. While uPA is inhibited by EGCG, we cannot find evidence in the literature that tPA is also inhibited. Furthermore, co-administration of tPA and EGCG to ischemic stroke patients extends the time window of the therapeutic tPA treatment, thus providing indirect evidence of tPA not being activated [91,92].

## 12. Conclusions

There is no remedy for Down syndrome, yet a recent clinical study [7] provides a glimmer of hope that EGCG might alleviate its symptoms. Although results presented in a Phase 2 clinical trial for individuals with DS are promising [7], there are other studies claiming no improvement [81,82,89,90] or presenting unwanted side effects [89] in the mouse models of DS [93,94,95,96] when EGCG was administrated.

Based on data available in the literature, we attempted to give a plausible explanation for how EGCG could slow down the abnormal degradation of NGF that is altered in this disease. The plasminogen activating system has an impact on the condition of neurons and neurites in Down syndrome. Its role is mainly connected with the delimitation of nerve growth factor deficiency. Diminished activity of tPA in Down syndrome brain converts less plasminogen to plasmin; this produces less proNGF to activate NGF. At the same time, plasmin activates proMMP-9. Activated MMP-9 inactivates the excess of NGF and the process is controlled by neurosepin, an endogenous inhibitor of tPA.

However, degradation of NGF can be simultaneously reduced by EGCG that inhibits MMP-9, which is strongly associated with uPA. Green tea is also a natural inhibitor of uPA. It is worth noting that tPA can also stimulate matrix metalloproteinases, especially MMP-9. The accumulated facts point to the possible protection of NGF by inhibition of MMP-9 by EGCG.

Interestingly, teaflavin inactivates PAI-1, which inhibits tPA. This is probably possible via brain tissue receptors such as PI3K/AKT, and MAPK, which are also abundant targets for EGCG.

Finally, some questions remain unanswered. For example, how can effects—if there are any—be imposed on the brain by EGCG metabolites or products of its degradation? Such doubts and questions can only be verified by well-designed preclinical and blind clinical studies on a sizable cohort and evaluated by a multidisciplinary team.
